# Craniofacial Asymmetry from One to Three Years of Age: A Prospective Cohort Study with 3D Imaging

**DOI:** 10.3390/jcm9010070

**Published:** 2019-12-27

**Authors:** Anniina M. Launonen, Ville Vuollo, Henri Aarnivala, Tuomo Heikkinen, Pertti Pirttiniemi, A. Marita Valkama, Virpi Harila

**Affiliations:** 1 Department of Oral Development and Orthodontics, Oulu University Hospital, 90220 Oulu, Finland; ville.vuollo@oulu.fi (V.V.); tuomo.heikkinen@oulu.fi (T.H.); pertti.pirttiniemi@oulu.fi (P.P.); virpi.harila@oulu.fi (V.H.); 2 Department of Oral Development and Orthodontics, Unit of Oral Health Sciences, Faculty of Medicine, University of Oulu, 90220 Oulu, Finland; 3 Medical Research Center Oulu, 90220 Oulu, Finland; henri.aarnivala@student.oulu.fi (H.A.); arjamaritavalkama@gmail.com (A.M.V.); 4 Department of Children and Adolescents, Oulu University Hospital, 90220 Oulu, Finland; 5 PEDEGO Research Group, 90220 Oulu, Finland

**Keywords:** facial asymmetry, craniofacial asymmetry, facial symmetry, deformational plagiocephaly, 3D stereophotogrammetry, three-dimensional, 3d-imaging, 3dMD, follow-up study, toddler, cohort study

## Abstract

Deformational plagiocephaly (DP) is considered a risk factor for facial asymmetry. This cohort-based, prospective, follow-up study used three-dimensional (3D) stereophotogrammetry to assess the development of facial asymmetry in a normal birth cohort and to investigate the impact of DP on facial asymmetry for the age range of one to three years. The study sample consisted of 75 children: 35 girls (47%) and 40 (53%) boys recruited from Oulu University Hospital. A total of 23 (31%) subjects had a history of DP in infancy. 3D facial images were obtained at the mean (SD) age of 1.01 (0.04) year old at T1 and 3.02 (0.14) years old at T2. To determine facial asymmetry, both landmark-based and surface-based facial symmetry methods were used. As measured with the surface-based methods, upper facial symmetry improved from T1 to T2 (*p* < 0.05). As measured with the landmark-based methods, facial symmetry improved on the upper and lower jaw from T1 to T2 (*p* < 0.05). The asymmetric effect of DP on the upper parts of the face tends to correct spontaneously during growth. Results indicate that previous DP does not seem to transfer to facial or occlusal asymmetry at the age of three years old.

## 1. Introduction

The human face is not perfectly symmetrical, and the amount of facial asymmetry present in a normal population varies depending on the study method used [1,2,3,4]. The etiology of increased facial asymmetry in childhood is suspected to rise from both hereditary and environmental factors [5,6]. Particular interest has been shown toward developmental factors such as harmful oral habits, unbalanced mastication side, constant facial pressure due to the unilateral sleeping position on one side [5,6], and occlusal abnormalities such as unilateral crossbite [7]. Reasons for mild directional facial asymmetries have been explored in studying the functional and structural differences between the cerebral hemispheres [8].

One suspected predisposing cause for facial asymmetry is deformational plagiocephaly (DP), a developmental, nonsynostotic, and acquired plagiocephaly. It is characterized as an asymmetrical head shape, which results from external pressure acting on the infant’s cranium. DP typically develops during the first months of life, and its highest prevalence is seen in the first six months of life [9]. DP has recently been reported concerning approximately one-third of infants in the European population, although the reported prevalence varies widely according to the study population and diagnostic method used [10,11]. DP is often included in the group of congenital factors of facial asymmetry [6], even though, based on the nature of the condition and its development postnatally, it can be classified among developmental factors of facial asymmetry.

In cross-sectional studies using cone-beam computer tomography, the DP is associated with facial asymmetry in infancy [12,13,14]. There appears to be a consensus that DP induces the anterior rotation of the cranial base and temporomandibular joint (TMJ) complex on the ipsilateral side [12,13,15], which may be the initial cause for facial asymmetries in those cases. Regarding mandibular and midfacial structures, there is still ambiguity about the role of these structures in DP-induced facial asymmetry. The authors of some studies report that the mandible is symmetrical and thus the detected facial asymmetry is mainly due to the rotation of the cranial base [12,15], while others have found a detectable shortening of mandibular and other facial structures on the affected side [13,14].

There is a lack of follow-up studies concerning facial asymmetries later in life among children with a history of DP. Mandibular deviation toward the unaffected side later in life has been associated with DP [16,17], but no comparison with a normal population has been made. Connections between a history of DP and malocclusion, lateral crossbite, or dental midline deviation in the primary dentition have been discussed. Occlusal abnormalities seem to be overrepresented among children with previous DP, even though the connection is not confirmed with statistical significance [17,18]. However, it has been reported that children with operated unilateral craniosynostosis in infancy have more facial asymmetry than controls [19]. Hence, it is assumed that a similar tendency, only milder, might be found among children with a history of DP.

Even though DP decreases throughout childhood, either spontaneously or with treatment, a notable amount of cranial asymmetry in the cases of DP can still be seen at the age of three to five years old [20]. Moreover, the growth potential of the upper and lower jaws is most significant during the first years of life [21,22]. Therefore, it is reasonable to assume that asymmetries in early childhood, such as DP, may lead to asymmetric growth in the facial area.

Traditionally, asymmetry has been studied using anthropometry [15], facial photographs [23], x-ray based orthopantomography [1], posteroanterior or oblique cephalograms [24], and cone-beam computed tomography [2]. With conventional methods, an obvious limitation arises from transferring a three-dimensional (3D) image to two dimensions, which increases possible errors. Other problems are the inaccuracies of landmark identification and the difficulty of defining the facial midline [23]. Furthermore, modern X-ray based longitudinal studies in a normal birth cohort are not feasible due to the potential risks of exposing children to ionizing radiation [25].

As if in response to problems faced with conventional methods, 3D facial imaging has been developed during the past few decades. 3D imaging enables a non-invasive, accurate method for analyzing facial asymmetry and following normal and disordered facial growth [26,27,28]. However, only a few longitudinal studies about the development of facial soft tissue asymmetry among healthy children have been published. In 3D studies of facial soft tissue asymmetry at the age of 5–10 years old [3] and 11–16 years old [4], facial asymmetry was not found to increase or decrease during the follow-up time.

The aim of this study was to longitudinally investigate the development of cranial and facial asymmetry from one to three years of age in a normal birth cohort. Of special interest was testing of the hypothesis that facial asymmetry at the age of one to three years has a relationship with DP in infancy.

## 2. Materials and Methods

### 2.1. Study Population and Study Design

**Figure 1:**
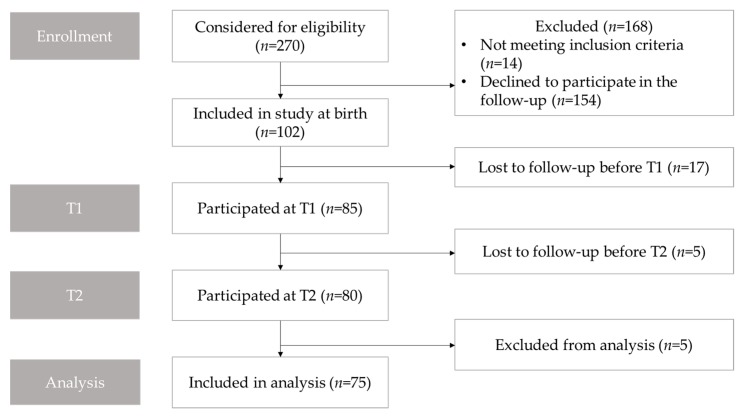
Flowchart showing participant enrollment, follow-up, and analysis. T1, the first follow-up approximately at the age of 12 months old; T2, the second follow-up approximately at the age of 3 years old.

This prospective, population-based cohort study was conducted in the Research Unit of Oral Health Sciences, the University of Oulu, and the Clinic for Children and Adolescents, Oulu University Hospital. All subjects had participated in previous studies concerning the development of DP in infancy [10,29,30,31], and all were re-invited to participate in this follow-up study (Figure 1).

Approval was obtained from the ethics committee of the Northern Ostrobothnia Hospital District (Oulu University Hospital; EETTMK 27/2011). Written informed consent was obtained from all of the parents. The study was registered in the National Clinical Trials register (NCT02283229).

Newborn infants born at Oulu University Hospital on pre-selected dates between February 2012 and December 2013 were recruited initially. A total of 102 newborns were enrolled in the study. Inclusion criteria were: children were born after 37 weeks of gestation, healthy enough to maintain without intensive care, and resided within 30 min driving distance from Oulu University Hospital. Exclusion criteria were diagnosed cheilopalatoshisis, craniosynostosis, or dysmorphic features.

For the previous study [10], subjects were randomized into two groups to test the impact of early parental educational guidance intervention on the prevalence and severity of DP. Parents of children in the interventional group (*n* = 35 (47%), in this study) received detailed recommendations regarding their infant’s environment, positioning, and handling before their discharge from the maternity ward. Full clarification of the enrollment process and detailed recommendations are reported in the context of the previous study [10]. 

### 2.2. Data Collection

Participants underwent examination and 3D stereophotogrammetric imaging of the head and face at the ages of three months, six months, 12 months, and three years old. Head shape and plagiocephaly were analyzed from every image. Facial images were analyzed when the study participants were approximately 12 months (T1) and three years (T2) of age.

3D images of the head and face were captured using a 3dMDhead^TM^ (Atlanta, GA, USA) 5-pod camera system. To prevent hair-induced disturbances in images, a tight nylon sock cap was fitted on each subject’s head, and all the hair from the forehead was set inside the cap, if possible. Subjects were seated on an adjustable chair at a standard distance from the cameras, and, if needed, a parent assisted each young child to optimally stabilize and center the head during imaging. Facial images were obtained in a natural head position, jaw relaxed [27]. Each facial image was evaluated immediately after capturing the image, and a new 3D-image was captured, if necessary.

Each participant’s dental occlusion was clinically examined at the age of three years. For sagittal occlusion, the anterior crossbite was registered, and the sagittal molar relationship on both sides was defined according to Angle classification. Sagittal occlusion of the molars was classified as neutral, mesial, or distal on the accuracy of half-cusp. For transversal occlusion, crossbite, scissor bite, and deviation (mm) of the mandibular dental midline from the facial midline was registered. Crossbite was registered if at least one maxillary posterior tooth had a buccal cusp occluding lingually to the buccal cusp of a mandibular tooth [32,33]. Occlusion was determined to be asymmetric if a subject had a deviation of dental midline one millimeter or greater, an asymmetric Angle classification between the right and left sides, or a unilateral crossbite.

Background data regarding pregnancy and delivery were collected from maternal and infant medical records. Infants’ parents filled out a questionnaire regarding care habits at each visit during the first year of follow-up.

### 2.3. Image Analysis

**Figure 2:**
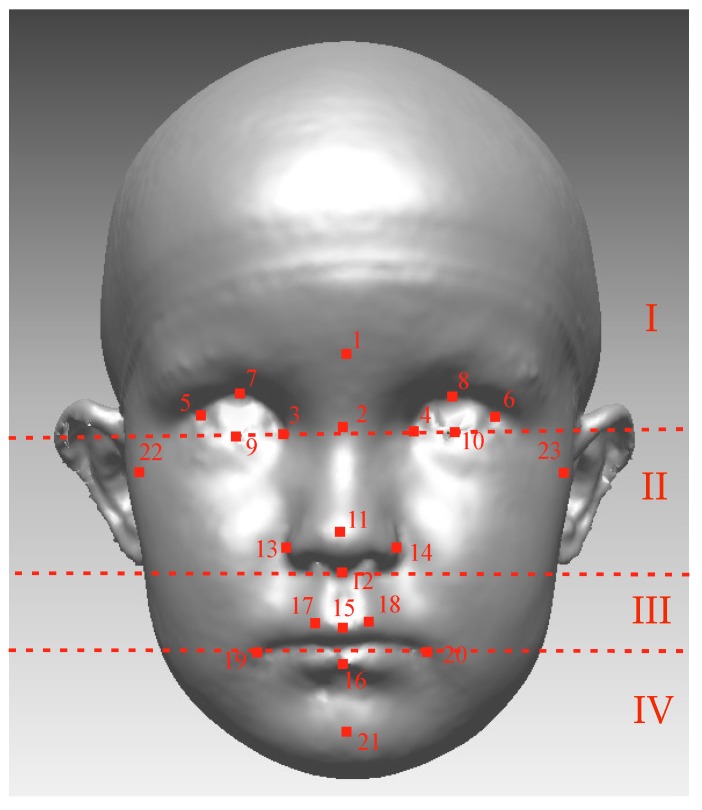
The Farcas soft-tissue landmarks: 1 Glabella (g); 2 Nasion (n); 3, 4 Endocanthion (en); 5, 6 Exocanthion (ex); 7, 8 Pulpabrale superius (ps); 9, 10 Pulpabrale inferius (pi); 11 Pronasale (prn); 12 Subnasale (sn); 13, 14 Alare (al); 15 Labiale superius (ls); 16 Labiale inferius (li); 17, 18 Christa philtra (cph); 19, 20 Cheilion (ch); 21 Pogonion (pg); and 22, 23 Tragion (Tr). Facial images were divided into four areas according to dashed lines: (**I**) the upper face (above the endocanthion line); (**II**) the upper mid-face (between the endocanthion line and the subnasal); (**III**) the lower mid-face (between the subnasal and the cheilion line); (**IV**) the lower face (under the cheilion line).

Rapidform2006 software (INUS Technology, Inc., Seoul, South Korea) was used to process and analyze 3D images. Twenty-three soft tissue landmarks [34,35] were identified manually for each image by one author (Figure 2). The facial position was standardized as proposed by Zhurov et al. [36]. After that, facial models were scaled based on the average centroid size (Frobenius norm of landmark matrix, in which landmark coordinates are in rows). All the position standardizing, scaling, and computation of the parameters were automated with a set of in-house Visual Basic for Applications (VBA) subroutines developed for Rapidform.

### 2.4. Cranial Asymmetry

To identify cranial asymmetry and to determine DP, the Oblique Cranial Length Ratio (OCLR) was calculated from the 3D image, and the cut-off point for DP was set at OCLR ≥ 104% as has been described in the context of our earlier study [10,37]. To illustrate location of the TMJ complex, the ear-offset (EO) was calculated as the difference between the right and left tragion along the z-axis.

### 2.5. Surface-Based Facial Symmetry Parameters

**Figure 3:**
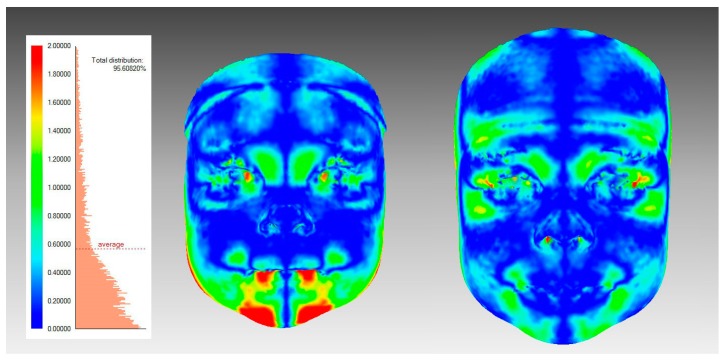
An illustration of surface-based facial symmetry analysis. A color-map is showing one participant’s facial asymmetry at T1 (left figure) and T2 (right figure); The colors demonstrate the deviations between the original and the mirrored facial shells.

To quantify facial asymmetry landmark-independently with surface-based methods (Figure 3), procedures were as follows: The widest possible face area was cut off from the 3D-image, using the rule that all distinct parts (like hair) have to be removed from each image. A mirrored 3D face was formed mirroring facial parts across the sagittal plane (YZ plane), and those two surfaces were superimposed using the best-fit technique on the facial area above the subnasal. The facial image was divided into the four following areas: the upper face (above the endocanthion line), the upper mid-face (between the endocanthion line and the subnasal), the lower mid-face (between the subnasal and the cheilion line), and the lower face (under the cheilion line) (Figure 2). The average distance (mm) between the original and mirrored face was calculated for the whole face and separately for all four facial areas. Further, the symmetry percentage was calculated as the face area where the distance between the original face and the mirrored surface did not exceed 0.5 mm. The used surface-based method and the chosen 0.5 mm limit for the symmetry percentage is previously described in the literature [3,4,38].

### 2.6. Landmark-Based Facial Symmetry Parameters

**Figure 4:**
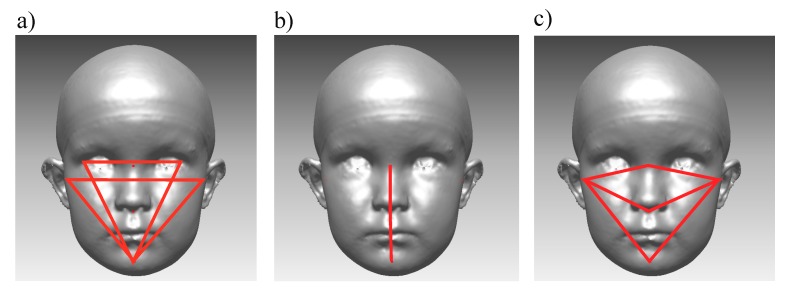
Landmark-based facial symmetry parameters. (**a**) Angles ExR-ExL-Pg, ExL-ExR-Pg, TrR-TrL-Pg, and TrL-TrR-pg; (**b**) Angle N-Sn-Pg; and (**c**) TrNa, TrSn, and TrPg lines.

Facial asymmetry was also analyzed using a landmark-based method with angular and linear measurements. First, the following angles were calculated both in 3D space and for their orthogonal projections onto the coronal plane (XY plane): Angles ExR-exL-Pg and ExL-exR-Pg (formed by a line connecting the exocanthions and pogonion) were measured and ExPg difference (the absolute value of the difference between ExR-ExL-Pg and ExL-ExR-Pg) was calculated from these angles. Angles TrR-TrL-Pg and TrL-TrR-Pg (formed by a line connecting the tragions and pogonion) were measured, and TrPg difference was formed respectively (Figure 4a) [4]. Both “ExPg difference” and “TrPg difference” describe lower jaw deviation, defined as the position of pogonion to exocanthions or tragions. The lower the value, the smaller the difference between angles, and thus, the more symmetrical the face is. Similarly, angle N-Sn-Pg on a coronal plane, and its absolute difference from 180° was calculated (Figure 4b).

In addition, relationships between landmark-based lines, measured in 3D space on the right and left sides of the face, were calculated from the images as follows: the Tragion-Nasion-ratio (TrNa ratio) as a relationship between the length of the left and right tragion-nasion lines; the Tragion-Subnasale-ratio (TrSn ratio), as a relationship between the length of left and right tragion-subnasale lines; and the Tragion-Pogonion-ratio (TrPg ratio), as a relationship between the length of the left and right tragion-pogonion lines. (Figure 4c). The relationship was calculated by dividing the left-side distance by the right-side distance. Thus, each ratio received a value higher than one, if the measured left side was longer than the corresponding right side. A similar relationship ratio was reported earlier [15,39]. Further on, for the TrNa, Tr-SN, and TrPg ratios, each of their absolute differences from 1 was calculated.

### 2.7. Subgroups

As a secondary outcome, to test the effect of DP on facial symmetry, subjects were divided into two subgroups based on the history of DP. A participant was classified in the “history of DP”-group if the participant had had DP (measured as OCLR > 104%) at the age of three months, six months, one year, or three years old. Also, the differences in facial symmetry parameters were tested between the following subgroups: early intervention group and control group; gender; and asymmetry in occlusion at the age of three years.

Also, as a secondary outcome, in order to test the connections of the side of EO and the side of the larger facial area in the whole study cohort, subjects were divided into two groups based on whether the right or left tragion was located sagittally more anteriorly. TrNa, TrSn, and TrPg ratios were compared between the two groups.

### 2.8. Statistical Analysis

For statistical work and data analyses, IBM SPSS Statistics version 25 was used. A paired sample *t*-test was used to determine the course of facial symmetry from T1 to T2. When analyzing the differences between subgroups, samples were relatively small, so the Shapiro-Wilk normality test was used to evaluate the normality. According to results from the tests, the normal distribution of the data could not be verified, so a non-parametric analysis (Mann-Whitney) was used to test the differences between subgroups at T1 and T2.

## 3. Results

Facial images were obtained from 85 subjects at T1, and 80 of those participated in the follow-up examination two years later at T2. Five subjects were excluded because their facial image was not acceptable due to the subject crying or moving during the imaging. Thus the final sample resulted in 75 cases screened and examined both at the age of one and three years (Figure 1). There were 35 girls and 40 (53%) boys in the final study sample. The mean (SD) age was 1.01 (0.04) years old at T1 and 3.02 (0.14) years old at T2.

### 3.1. The Primary Outcome

The course of facial symmetry from T1 to T2 using the surface-based method is shown in Table 1. The average distance decreased from the age of one year (T1) to the age of three years (T2) in every area of the face except for the lower midface. This change was statistically significant for the upper face only (*p* = 0.024). There was an increasing tendency for symmetry percentage from T1 to T2 for the whole face, upper face, and lower face and a decreasing tendency for mid-face. However, no statistical significance was reached (Table 1).

Changes in landmark-based parameters from T1 to T2 are presented in Table 2. Landmark-based linear parameters, TrSn and TrPg, showed significant improvement of facial symmetry between T1 and T2 on the upper and lower jaw.

### 3.2. Subgroups

No statistically significant difference was noted at T1 or T2 in any symmetry parameters between the subgroups describing genders, intervention versus control group, or the presence of an asymmetric occlusal trait.

### 3.3. Deformational Plagiocephaly

A total of 23 (31%) out of 75 subjects had a history of DP measured as OCLR > 104%. In the study population, the incidence of DP decreased gradually during the observation period: 18 (24%) subjects had DP at the age of 3 months old; 15 (20%) subjects had DP at the age of six months old; 12 (16%) subjects had DP at the age of one year old, and 9 (12%) subjects had DP at the age of three years old. Facial symmetry parameters compared with the history of DP are presented in Table 3. At T1, there were significantly higher average distances between the original and mirrored facial models in the DP group on the upper face (*p* = 0.022) and upper midface (*p* = 0.008). The difference between the two groups diminished from T1 to T2, and at T2, there was a significant difference left only on the upper face (*p* = 0.042) (Table 3). No statistically significant difference was noted between the DP group and controls in any of the angular or linear symmetry parameters.

### 3.4. Side of the DP Related to Facial Asymmetry

In the DP group, the right side was the affected side in a total of 15 cases, and in 8 cases the left side was the affected side. At T1, the anterior EO was on the affected side in 9 cases and on the unaffected side in 14 cases. AT T2, the anterior EO was on the affected side in 12 cases and on the unaffected side in 11 cases. A connection between the side of the DP and the anterior EO on the affected side could not be found.

When the side of the previous DP was compared to linear facial symmetry parameters, measured in 3D space, the unaffected side was found significantly larger than the affected side of DP both at T1 and T2 (Table 4).

### 3.5. Ear-Offset and Facial Asymmetry

Overall, in the whole study sample, the anterior EO was more common on the left side than on the right side of the face; at T1, 58 (77%) subjects had the left tragion located more anteriorly, and 17 subjects had the right tragion located more anteriorly. At T2, 54 (72%) subjects had the left tragion located more anteriorly, and 21 subjects had the right tragion located more anteriorly. Table 5 represents the relationship of TrNa, TrSn, and TrPg ratios to the side of the anterior EO at T1 and T2. At both timepoints, the ratios were greater if the right tragion was located anteriorly and smaller if the left tragion was located anteriorly, indicating larger facial measurements on the contralateral side of the anterior EO. The differences were statistically significant at both T1 and T2 (Table 5).

### 3.6. Occlusal Parameters

Occlusal parameters were analyzed at the age of three years old. One participant had total anodontia and was therefore excluded from the analysis. The sagittal relationship was not recorded from four subjects because of a lack of cooperation. There were no statistically significant differences in occlusal parameters at the age of three years old between the DP group and the control group. Six subjects from the DP group had a deviation of the mandibular midline, and four of those were toward the affected side while two were toward the unaffected side (Table 6).

## 4. Discussion

This longitudinal, three-dimensional study was created to investigate the course of facial symmetry and to assess the impact of DP on facial asymmetry at the ages of one and three years. We chose to analyze facial symmetry using both the landmark-based and the surface-based approach.

Results from this study indicate facial asymmetry is already present at the age of one to three years. As measured with surface-based methods, using the average distance and the symmetry percentage, facial asymmetry in our study subjects did not tend to change during growth on the mid and lower face. On the upper face (above the eyes), there was a statistically significant improvement of facial symmetry. In the case of mid-face and lower face, our results are analogous to those of Primozic et al. [3], although the participants were younger in this study. Primozic et al. followed 27 subjects from ages 5–10 years old using similar surface-based analyzing methods and found facial asymmetry already present at the age of five years remaining unchanged after that. However, the improvement of upper face symmetry found in this study was not reported by Primozic et al.

In this normal birth cohort, the left tragion was found to be located anteriorly more often than the right one, and linear facial parameters were found to be significantly longer on the right side if the left tragion was more anterior and vice-versa. Our results support those from previous studies relevant to the relationship between facial structures and the asymmetry of the neurocranium and cranial base [40,41,42,43]. Pirttiniemi et al. [40] studied dry human Lapp skulls and found the right glenoid fossa located more laterally and distally than the left. They found a similar correlation with cranial base and facial asymmetry. Also in their study, on the side of the distally located glenoidal fossa, the mandible was increased in length. Heikkinen et al. [41,42,43] studied occlusal facets and malocclusion asymmetry prevalences in true right-sided and nonright-sided children and found connections between the neurocranium and the structures supporting occlusion. According to Pirttiniemi, the underlying cause for slight directional asymmetry might be the anatomical and functional differences of the right and left hemispheres [8]. In previous studies, conducted with subjects at an older age, a significant aspect has been the possible involvement of functional factors. In the recent study, the left-right differences were already found at the age of one to three years. However, during this age range, primary teeth are erupting into occlusion, and a variety of factors (tooth eruption order, dental morphology, feeding type, and food quality) may have effects on occlusal function.

In this normal birth cohort, 23 out of 75 children had a history of DP in early childhood. In the study sample, the expected connection between the side of the DP and the anterior EO on the affected side was not found. However, the plagiocephaly-induced rotation of the cranial base and anterior shifting of the TMJ complex has been demonstrated in earlier studies using a cone-beam computed tomography [12,13].

According to the present study, the DP seems to affect facial asymmetry at the age of one year as subjects with current DP or a history of DP had significantly more asymmetry on their whole face, upper face, and upper midface. However, the asymmetrical effect seems to diminish mostly during growth from one year to three years; at the age of three years, there was a statistically significant difference between the two groups only on the upper face. To our knowledge, no previous study has used a surface-based 3D method to study the effect of DP on facial asymmetry after infancy.

In the literature, there are conflicting reports about how the side of the DP affects facial asymmetry. Moon et al. and Netherway et al. [13,14] used cone-beam computed tomography and reported significant asymmetry in mandibular measurements in children with DP. On the affected side, mandibular body length was statistically significantly shorter. Moon et al. further found that facial asymmetry was more significant in patients with DP than in controls. St. John et al. and Smartt et al. found controversial results [12,15]. St John et al. used anthropometry to analyze infants at six months of age and recognized mandibular parameters not to be significantly different between affected and unaffected sides in patients with DP. Smartt et al. found the same result from a study in which they used 3D computed tomography; they also reported that the mandible itself was symmetric, but the articular fossa is located more posteriorly on the unaffected side [12]. All of those studies used landmark-based methods and measured facial asymmetry cross-sectionally in subjects presenting with DP.

In our study, the linear facial parameters TrNa, TrSn, and TrPg were larger on the unaffected side of the face than on the affected side, and our results thus support the findings of Moon and Netherway. However, in this study, there was not more facial asymmetry in the DP group than in the control group when measured with landmark-based parameters. Moreover, in our whole study cohort, facial symmetry improved from one to three years of age for the upper and lower jaw according to landmark-based measurements. Kreuz et al. conducted a longitudinal study on facial asymmetry correction from 3D images using the same landmark-based measurements and reported that facial symmetry improved with helmet therapy treatment during their follow-up time of five months [39]. None of the subjects in our study cohort had a severe form of DP, and no helmet treatment was needed for anyone. In addition, the follow-up time in our study was longer than that of Kreuz et al., and the development of facial asymmetry was analyzed in a normal birth cohort, which might explain the differences found.

In this study population, there was only one lateral crossbite. Thus, the incidence of crossbites in this study sample is smaller than previously reported; in Swedish studies, the incidence of lateral crossbites at the age of three years old is more than 10% [44,45]. In the DP group, no connections were found with the side of DP and the side of occlusal asymmetries, such as midline deviation. Thus our result does not support earlier findings that the majority of occlusal deviations are toward the contralateral side of occipital flattening [17,46]. According to our study, DP in infancy does not seem to cause occlusal asymmetries or asymmetry of the lower jaw at the age of three years old, and the asymmetric effect on the upper face seems to diminish during growth from one to three years of age.

The strengths of this study include its longitudinal, follow-up study design; its cohort-based study population; and its use of 3D stereophotography. This method, soft-tissue 3D stereophotography, is a modern tool for analyzing facial growth [26] and facial symmetry [47,48,49]. As a photography-based method, it is non-invasive and thus suitable for longitudinal studies [3,4]. 3D imaging is proven to be highly reliable and repeatable, even using landmark-based analysis [28]. The geometric accuracy of 3dMD^TM^ is < 0.2 mm, and reproducibility of 3D images is reported to be as low as 0.17 mm [50]. The landmark-based method also reaches quite a high reproducibility: Intra-examiner reproducibility has been reported to be less than 1 mm in half of the cases, and inter-examiner even less [51]. The accuracy ranges from 0.39 to 1.49 mm, depending on the chosen landmark [35].

One possible limitation of this study could be the use of soft tissue imaging. However, according to recent research with cone-beam computed tomography, facial soft tissue has a significant relationship with facial skeletal shape and asymmetry, and thus facial symmetry analyzed from soft tissue can be considered reliable [52,53].

Method inaccuracies became more apparent when measuring differences of very short distances as EO, which might explain why anterior EO on the affected side of DP could not be found in this study. Accuracy of the lower jaw landmarks is naturally dependent on each subject’s ability to hold the lower jaw in a stable position during imaging, even though the 3D-images were obtained jaw relaxed in natural head position, and each facial shell was evaluated after capturing the image. Since this study is cohort-based, the number of subjects with a history of DP remains quite low. As a result of the limited number of occlusal abnormalities, we could not determine the connection between the side of DP and the side of asymmetric occlusion. There is still a need for a larger study sample of children with DP to confirm the connections with DP diagnosed at an early age and the later need for orthodontic treatment. In addition, because of multiple comparisons in the recent study, the family-wise errors can occur, and therefore, further investigation is suggested.

Further research should also focus on the use and applications of 3D imaging. Particular attention should be paid to recognizing facial asymmetry and other disturbances in normal facial growth, using 3D images. Developing the diagnostic methods and the software of 3D imaging would help with the early recognition of patients that need more precise follow-up but also address orthodontic professionals to optimize treatment timing.

## 5. Conclusions

Upper facial symmetry improved from one to three years of age in a normal birth cohort as measured with a surface-based approach. As measured with landmark-based methods, facial symmetry improved on the mid-face and lower face between one and three years. The asymmetric effect of DP on the upper parts of the face tends to correct spontaneously during growth between the ages of one and three years. Previous DP does not seem to transfer to facial or occlusal asymmetry at the age of three years.

## Figures and Tables

**Table 1:** The course of facial symmetry measured with surface-based variables: average distance (mm) and symmetry percentage (%) at T1 and T2. SD = Standard deviation; IQR = Inter quartile range; Bold values denote statistical significance at the *p* < 0.05 level (a = Paired sample *t*-test was used).

Part of the Face	T1			T2			*p* ^a^
	Mean (SD)	Median	IQR	Mean (SD)	Median	IQR	
Average Distance (mm)							
Whole face	0.52 (0.17)	0.48	0.41–0.62	0.49 (0.13)	0.47	0.41–0.58	0.160
Upper face	0.45 (0.16)	0.42	0.33–0.55	0.41 (0.16)	0.37	0.30–0.50	**0.024**
Upper mid face	0.48 (0.21)	0.43	0.31–0.55	0.46 (0.18)	0.44	0.30–0.58	0.312
Lower mid face	0.56 (0.24)	0.51	0.41–0.68	0.58 (0.22)	0.56	0.40–0.75	0.478
Lower face	0.75 (0.40)	0.65	0.48–0.87	0.66 (0.36)	0.55	0.36–0.88	0.130
Symmetry Percentage (%)							
Whole face	63.3 (10.7)	63.5	56.9–70.4	64.4 (10.5)	65.1	58.7–69.9	0.444
Upper face	67.8 (13.6)	70.0	60.4–75.8	70.9 (14.2)	72.1	61.4–82.5	0.059
Upper mid face	68 (13.1)	66.7	59.9–77.1	67.9 (14.5)	66.7	56.9–81.3	0.945
Lower mid face	59.9 (16.7)	58.1	50.1–70.4	56.0 (17.5)	55.0	45.2–70.7	0.177
Lower face	46.3 (18.7)	48.5	32.6–61.4	51.2 (23.4)	52.3	29.5–71.1	0.131

**Table 2:** The course of facial symmetry measured with surface-based variables at T1 and T2. 3D: three-dimensional; XY, coronal plane; SD = Standard deviation; IQR = Inter quartile range; Bold values denote statistical significance at the *p* < 0.05 level (a = Paired sample *t*-test was used).

	T1			T2			*p* ^a^
	Mean (SD)	Median	IQR	Mean (SD)	Median	IQR	
ExPg difference 3D (°):	1.45 (1.28)	1.09	0.46–2.09	1.13 (0.95)	0.85	0.33–1.57	0.095
ExPg difference XY (°):	1.43 (1.21)	1.18	0.50–1.95	1.10 (0.96)	0.92	0.31–1.71	0.066
TrPg difference 3D (°):	1.39 (0.94)	1.24	0.72–2.02	1.12 (0.82)	0.99	0.40–1.69	**0.040**
TrPg difference XY (°):	1.01 (0.92)	0.86	0.39–1.41	0.99 (0.82)	0.87	0.28–1.37	0.897
Difference of N-Sn-Pg from 180°, XY (°)	1.38 (1.2)	1.04	0.53–1.85	1.37 (1.07)	1.15	0.53–1.95	0.948
Absolute value of difference between TrNa Ratio and 1	0.019 (0.012)	0.018	0.011–0.027	0.017 (0.013)	0.013	0.006–0.024	0.173
Absolute value of difference between TrSn Ratio and 1	0.019 (0.014)	0.017	0.008–0.029	0.020 (0.010)	0.014	0.006–0.023	**0.032**
Absolute value of difference between TrPg Ratio and 1	0.017 (0.012)	0.015	0.009–0.026	0.010 (0.010)	0.011	0.005–0.019	**0.010**
Absolute value of EO	1.82 (1.22)	1.51	0.85–2.78	1.55 (1.10)	1.32	0.67–2.32	0.124

**Table 3:** Comparison of facial symmetry parameters between the deformational plagiocephaly (DP) group and the controls; Average distance (mm) and symmetry percentage (%) at the age of one year old (T1) and three years old (T2). SD = Standard deviation; IQR = Inter quartile range; Bold values denote statistical significance at the *p* < 0.05 level (b = Mann-Whitney nonparametric test was used).

Part of the Face	No History of DP (*n* = 52)			History of DP (*n* = 23)			*p* ^b^
	Mean (SD)	Median	IQR	Mean (SD)	Median	IQR	
T1							
Average distance (mm)							
Whole face	0.49 (0.16)	0.46	0.39–0.56	0.59 (0.17)	0.54	0.46–0.66	**0.014**
Upper face	0.43 (0.16)	0.39	0.32–0.48	0.51 (0.16)	0.48	0.33–0.59	**0.022**
Upper mid-face	0.43 (0.16)	0.41	0.31–0.51	0.58 (0.26)	0.53	0.43–0.72	**0.008**
Lower mid-face	0.53 (0.23)	0.50	0.36–0.64	0.62 (0.26)	0.57	0.45–0.74	0.121
Lower face	0.74 (0.39)	0.64	0.47–0.86	0.78 (0.42)	0.69	0.48–0.99	0.890
Symmetry percentage (%)							
Whole face	64.7 (11.4)	65.6	59.1–72.6	60.1 (8.5)	62.6	54.8–65.4	0.060
Upper face	69.4 (14.1)	71.0	63.5–77.1	64.2 (11.9)	66.8	53.8–74.2	0.091
Upper mid-face	69.9 (13.3)	68.2	61.6–80.6	63.8 (11.8)	64.8	53.6–72.5	**0.049**
Lower mid-face	61.5 (17.7)	62.3	52.1–74.2	56.1 (13.7)	54.3	45.4–67.3	0.175
Lower face	46.0 (17.8)	43.1	32.7–61.2	47.0 (21.0)	50.6	28.2–63.8	0.613
T2							
Average distance (mm)							
Whole face	0.48 (0.13)	0.45	0.41–0.56	0.52 (0.14)	0.51	0.40–0.64	0.396
Upper face	0.38 (0.13)	0.34	0.29–0.46	0.48 (0.19)	0.44	0.32–0.56	**0.042**
Upper mid-face	0.43 (0.15)	0.43	0.30–0.53	0.52 (0.21)	0.52	0.35–0.68	0.093
Lower mid-face	0.59 (0.24)	0.51	0.38–0.76	0.57 (0.17)	0.61	0.45–0.69	1.000
Lower face	0.70 (0.37)	0.59	0.41–0.91	0.58 (0.31)	0.47	0.32–0.75	0.138
Symmetry percentage (%)							
Whole face	64.7 (10.6)	65.7	58.2–70.0	63.9 (10.5)	64.4	59.3–69.9	0.890
Upper face	72.6 (14.3)	76.4	61.7–82.9	67.0 (13.7)	68.7	59.6–79.5	0.100
Upper mid-face	68.3 (14.9)	67.0	57.4–81.7	67.1 (14.0)	62.6	56.5–77.6	0.671
Lower mid-face	55.1 (18.7)	54.6	40.6–71.1	58.1 (14.7)	55.5	47.9–66.3	0.550
Lower face	48.5 (23.1)	48.8	28.5–70.0	57.2 (23.3)	59.8	31.4–80.8	0.121

**Table 4:** Linear symmetry parameters (TrNa ratio, TrSN ratio, and TrPg ratio) at T1 and T2 and the difference between the following subgroups: Right side as an affected side of DP and left side as an affected side of DP. SD = Standard deviation; IQR = Inter quartile range; Bold values denote statistical significance at the *p* < 0.05 level (b = Mann-Whitney nonparametric test was used).

	Right-Sided DP (*n* = 15)			Left-Sided DP (*n* = 8)			Mean Difference	*p* ^b^
	Mean (SD)	Median	IQR	Mean (SD)	Median	IQR		
T1								
TrNa ratio	1.012 (0.015)	1.012	1.000–1.026	0.981 (0.026)	0.975	0.958–1.010	0.031	**0.011**
TrSn ratio	1.007 (0.016)	1.009	0.998–1.016	0.979 (0.027)	0.973	0.957–1.004	0.028	**0.005**
TrPg ratio	1.002 (0.024)	0.995	0.989–1.017	0.987 (0.020)	0.987	0.969–1.005	0.015	0.146
T2								
TrNa ratio	1.007 (0.012)	1.012	0.994–1.016	0.980 (0.017)	0.980	0.964–0.995	0.027	**<0.001**
TrSn ratio	1.000 (0.013)	1.001	0.991–1.014	0.977 (0.016)	0.975	0.966–0.989	0.023	**0.001**
TrPg ratio	0.998 (0.013)	0.998	0.986–1.004	0.980 (0.008)	0.981	0.973–0.985	0.018	**0.002**

**Table 5:** Linear symmetry variables grouped by the side of the ear-offset (EO) at T1 and T2. SD = Standard deviation; IQR = Inter quartile range; Mann-Whitney nonparametric test was used to calculate *p*-values. Bold values denote statistical significance at the *p* < 0.05 level.

	Right Tragion Anteriorly			Left Tragion Anteriorly			*p*
	Mean (SD)	Median	IQR	Mean (SD)	Median	IQR	
T1	T1 (*n* = 17)			T1 (*n* = 58)			
TrNa ratio	1.017(0.012)	1.013	1.008–1.027	0.986(0.019)	0.983	0.974–0.998	**<0.001**
TrSN ratio	1.013(0.012)	1.013	1.003–1.020	0.983(0.018)	0.983	0.968–0.996	**<0.001**
TrPo ratio	1.006(0.020)	1.012	0.992–1.015	0.987(0.016)	0.984	0.975–0.999	**<0.001**
T2	T2 (*n* = 21)			T2 (*n* = 54)			
TrNa ratio	1.017(0.019)	1.016	0.999–1.024	0.988(0.016)	0.989	0.975–1.000	**<0.001**
TrSN ratio	1.010(0.018)	1.008	0.997–1.019	0.985(0.013)	0.986	0.976–0.994	**<0.001**
TrPo ratio	1.003(0.017)	1.003	0.991–1.016	0.990(0.012)	0.990	0.981–1.001	**0.002**

**Table 6:** Occlusal parameters at the age of three years old (T2), history of DP compared to no history of DP.

	History of Deformational Plagiocephaly			
	NO (*n* = 51)		YES (*n* = 23)	
	N	%	N	%
Asymmetric occlusal trait				
Deviation of mandibular midline	8	16%	6	26%
Asymmetric molar relationship	4	8%	1	4%
Crossbite	1	2%	0	0%
Total	11	22%	7	30%
Sagittal relationship				
Normal	35	73%	16	73%
Distal	8	17%	4	18%
Mesial	1	2%	1	4.5%
Asymmetric	4	8%	1	4.5%
